# *Drosophila* S2 Cells Are Non-Permissive for Vaccinia Virus DNA Replication Following Entry via Low pH-Dependent Endocytosis and Early Transcription

**DOI:** 10.1371/journal.pone.0017248

**Published:** 2011-02-15

**Authors:** Zain Bengali, P. S. Satheshkumar, Zhilong Yang, Andrea S. Weisberg, Nir Paran, Bernard Moss

**Affiliations:** Laboratory of Viral Diseases, National Institute of Allergy and Infectious Diseases, National Institutes of Health, Bethesda, Maryland, United States of America; University of Cambridge, United Kingdom

## Abstract

Vaccinia virus (VACV), a member of the chordopox subfamily of the *Poxviridae*, abortively infects insect cells. We have investigated VACV infection of *Drosophila* S2 cells, which are useful for protein expression and genome-wide RNAi screening. Biochemical and electron microscopic analyses indicated that VACV entry into *Drosophila* S2 cells depended on the VACV multiprotein entry-fusion complex but appeared to occur exclusively by a low pH-dependent endocytic mechanism, in contrast to both neutral and low pH entry pathways used in mammalian cells. Deep RNA sequencing revealed that the entire VACV early transcriptome, comprising 118 open reading frames, was robustly expressed but neither intermediate nor late mRNAs were made. Nor was viral late protein synthesis or inhibition of host protein synthesis detected by pulse-labeling with radioactive amino acids. Some reduction in viral early proteins was noted by Western blotting. Nevertheless, synthesis of the multitude of early proteins needed for intermediate gene expression was demonstrated by transfection of a plasmid containing a reporter gene regulated by an intermediate promoter. In addition, expression of a reporter gene with a late promoter was achieved by cotransfection of intermediate genes encoding the late transcription factors. The requirement for transfection of DNA templates for intermediate and late gene expression indicated a defect in viral genome replication in VACV-infected S2 cells, which was confirmed by direct analysis. Furthermore, VACV-infected S2 cells did not support the replication of a transfected plasmid, which occurs in mammalian cells and is dependent on all known viral replication proteins, indicating a primary restriction of DNA synthesis.

## Introduction

The *Poxviridae*, a family of large enveloped double-stranded DNA viruses that replicate exclusively in the cytoplasm, are divided into chordopox and entomopox subfamilies, which infect vertebrate and invertebrates respectively [1]. Approximately 100 genes are conserved in all chordopoxviruses and about half of these can be identified in entomopoxviruses [2]. Although host restrictions prevent the complete replication of chordopoxviruses in insect cells and entomopoxviruses in mammalian cells, viral protein synthesis has been detected under non-permissive conditions [3]. *Amsacta moorei*, the prototypic entomopoxvirus, expresses only early genes in vertebrate cells [4]. Some viral early and late gene expression as well as DNA replication occur in gypsy moth cells infected with vaccinia virus (VACV), the prototypic chordopoxvirus that was used for smallpox eradication, but assembly of virus particles fails to occur [5]. In VACV-infected *Drosophila* S2 cells, only expression of the beta-galactosidase reporter gene regulated by an early promoter was detected [6], suggesting variations in the extent to which insect cells can support chordopoxvirus replication.

The nearly 200-kbp double-stranded genome of VACV codes for approximately 200 proteins with roles in entry, RNA and DNA synthesis, morphogenesis, and host interactions [1]. The VACV replication cycle begins with the attachment of virions to glycosaminoglycans or laminin on the cell surface [7], [8], [9], [10] and entry proceeds concurrently by fusion of the viral membrane with the plasma membrane and with low-pH vesicle membranes following endocytosis, with the extent that each pathway is used depending on the virus strain and cell type [11], [12], [13]. The entry-fusion complex (EFC), comprised of more than 10 viral membrane proteins, is required for infection [14], [15], [16], [17], [18], [19], [20], [21], [22], [23], [24], [25]. Entry of the core, containing a complete transcription system, into the cytoplasm results in the production of more than 100 early mRNA species [26]. The early mRNAs encode proteins required for replication of the genome and transcription of intermediate stage mRNAs. Following DNA replication, intermediate mRNAs encoding late transcription factors and late mRNAs encoding virion components are synthesized and translated within cytoplasmic factories, where virions are then assembled [27]. The infectious mature virion (MV) consists of a nucleoprotein core surrounded by a single membrane bilayer containing about 20 viral proteins [28]. Most of the MVs remain in the cell until lysis; however, a subset are wrapped with modified Golgi or endosomal membranes, transported to the periphery and released from the interior of the cell by exocytosis for efficient cell-to-cell spread [29].

Poxviruses are generally considered to be more self-sufficient than many other viruses owing to their large genomes and cytoplasmic site of replication. In this context, we were motivated to further investigate the inability of VACV to complete its replication cycle in insect cells. Our interest was further stimulated by the availability of powerful tools for genetic analysis, particularly the robust RNAi system for *Drosophila* [30]. An RNAi kinome (∼440 genes encoding protein kinases, phosphatases and regulatory factors) screen of VACV infection of *Drosophila* cells identified AMP-activated kinase as an essential entry factor that is conserved in mammals [6]. In order to more fully exploit the *Drosophila* system for genome-wide screens, it is important to further characterize the replication block for VACV. Here we showed that VACV entry into a *Drosophila* S2 cell depended on the EFC but appeared to occur exclusively by a low pH-dependent endocytic mechanism, in contrast to the dual entry pathways used in mammalian cells. Deep RNA sequencing revealed that the entire VACV early transcriptome was expressed. Nevertheless, replication of the viral genome and late stage mRNA and proteins were not detected. Replication of a transfected plasmid also failed to occur, indicating a block beyond uncoating of the virus core. In addition, the synthesis of post-replicative stage proteins could be overcome by transfecting plasmids with intermediate and late promoters, consistent with a primary block in DNA replication.

## Results

### Entry of VACV into S2 cells requires components of the EFC

**Figure 1:**
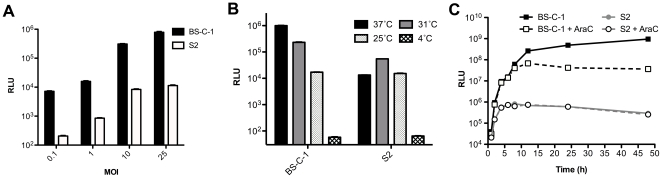
Entry of VACV in *Drosophila* S2 cells. **A**) BS-C-1 and S2 cells were incubated with purified WRvFire virions at 4°C at neutral pH for 1 h at the indicated MOI to allow attachment, washed, and then incubated for 1 h at 37°C for BS-C-1 cells and 25°C for S2 cells. Cells were lysed and Luc activity measured and plotted as relative light units (RLU). **B**) Cells were infected as in panel A with a MOI of 2 PFU per cell and after virus attachment the plates were incubated at the indicated temperatures for 2 h and then assayed for Luc. **C**) Cells were infected with an MOI of 1 PFU per cell as in panel A. After attachment, plates were incubated at 31°C and Luc assays were made over a 48 h period. Note that the solid and dashed lines representing Luc activity from S2 cells in the presence and absence of AraC are practically superimposed. Standard error bars were plotted in all three panels but are too close to discern in some places.

We used a recombinant VACV strain WR containing the firefly Luc gene regulated by the synthetic early/late promoter to determine entry unless otherwise stated. *Drosophila* S2 and African green monkey BS-C-1 cells were infected with various amounts of virus and Luc activity was measured after 1 h (Fig. 1A). Activity was proportional to the multiplicity of infection but was about 2-logs higher in BS-C-1 than S2 cells. Since BS-C-1 and S2 cells are grown and maintained at 37°C and 25°C, respectively, we tested the effects of temperature. Whereas Luc activity increased from 25 to 31 to 37°C in BS-C-1 cells, the S2 cells had maximal Luc activity at 31°C (Fig. 1B). In subsequent experiments, both BS-C-1 and S2 cells were incubated at the compromise temperature of 31°C following virus attachment.

To determine whether the relatively low Luc expression in S2 cells was due to slower kinetics, activity was measured over a 48 h period in the presence or absence of AraC, an inhibitor of DNA replication. Under the former condition, only early gene expression could occur. In S2 cells, expression reached a plateau at 6 h in the presence or absence of inhibitor (Fig. 1C), suggesting that only early gene expression occurred under both conditions. In contrast, Luc expression continued for more than 24 h in BS-C-1 cells in the absence of AraC but plateaued earlier in the presence of the inhibitor (Fig. 1C), consistent with early and late gene expression under the former conditions. Taken together, the data indicated that either entry or subsequent gene expression of VACV was lower in S2 cells than in mammalian cells and suggested that only early gene expression occurred in S2 cells.

**Figure 2:**
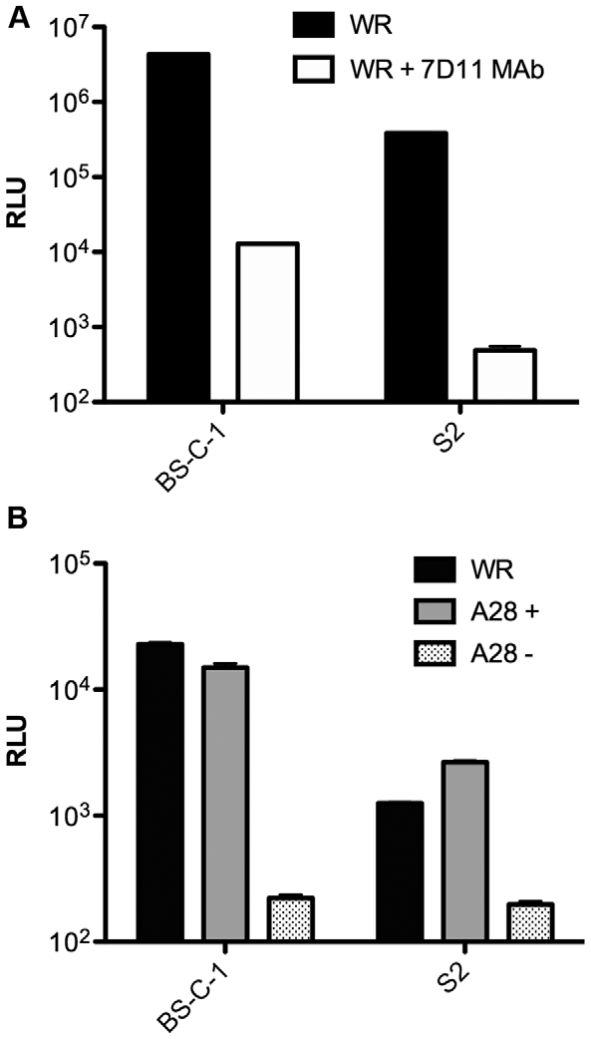
VACV entry into S2 cells is dependent on components of the EFC. **A**) Purified WRvFire virions (WR) (MOI of 10 PFU per cell) were incubated with or without the L1 MAb 7D11 (20 µg/ml) for 30 min at room temperature and then added to BS-C-1 or S2 cells. After attachment, the cells were incubated at 31°C for 1 h and assayed for Luc. **B**) BS-C-1 and S2 cells were incubated with purified WRvFire virions (WR) or A28ivFire virions (MOI of 10 PFU per cell) containing (+) or lacking (-) the A28 protein. After attachment, the cells were incubated for 1 h and assayed for Luc. Standard error bars were plotted in both panels but are too close to discern in some places.

To investigate whether entry of VACV into S2 cells occurs by mechanisms used in mammalian cells, we determined the effects of the neutralizing MAb 7D11 targeted to the L1 entry protein. As shown in Fig. 2A, VACV WR entry was inhibited by MAb 7D11 in both BS-C-1 and S2 cells. To more directly assess the role of the EFC, a recombinant VACV with the gene encoding the A28 EFC protein regulated by the *Escherichia coli lac* repressor and expressing Luc was constructed. Infectious and non-infectious virions were prepared by infecting BS-C-1 cells in the presence and absence of the inducer IPTG as previously described [14]. The virions made in the presence of IPTG contained A28 whereas those made in the absence of IPTG lacked A28. Entry of virions lacking A28 was severely inhibited in both cell types (Fig. 2B). We concluded that components of the VACV entry fusion complex are required for entry in S2 cells.

### Entry of VACV into S2 cells occurs by low pH-dependent endocytosis

**Figure 3:**
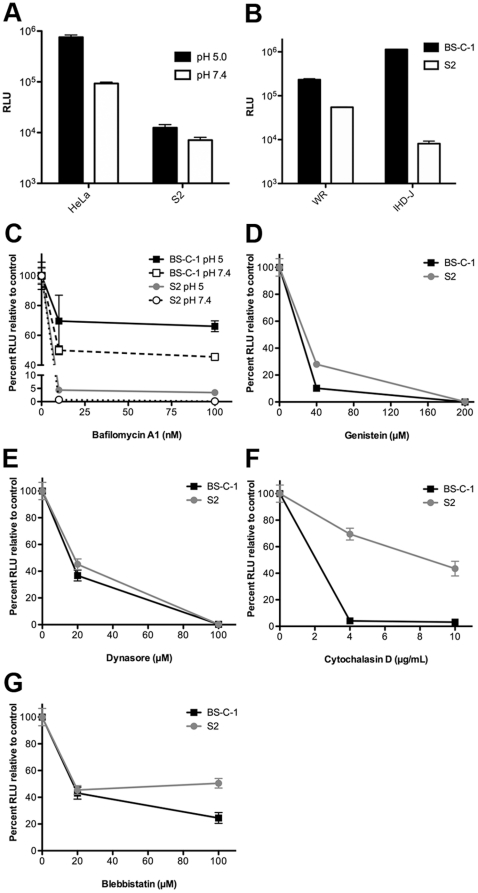
Effects of pH and chemical inhibitors on VACV entry. **A**) HeLa and S2 cells were incubated with purified WRvFire virions at 4°C for 1 h at a MOI of 2 PFU per cell. After attachment, cells were washed and incubated with pH 5 or pH 7.4 buffer for 3 min at 37°C. Cells were then washed and incubated at 31°C at neutral pH for 2 h and Luc assayed. **B**) BS-C-1 and S2 cells were infected with WRvFire or IHD-JvFire virions at neutral pH and Luc assayed as in panel A. **C**) BS-C-1 and S2 cells were infected as above except cells were pre-treated with bafilomycin A1 for 1 h at 31°C and then pre-chilled to 4°C prior to virion attachment, followed by wash and pH treatment. Inhibitor was maintained in the media throughout the infection. BS-C-1 and S2 cells were pretreated with: **D**) genistein; **E**) dynasore; **F**) cytochalasin; **G**) blebbistatin followed by wash and incubation at 31°C for 2 h. Standard error bars were plotted in all panels but are too close to discern in some places.

Entry of VACV strain WR into mammalian cells occurs by two routes simultaneously: direct fusion with the plasma membrane and low pH-dependent endocytosis. The latter pathway was established by showing that brief low pH treatment accelerated entry through the plasma membrane by mimicking the pH of endosomes and by preventing endosomal acidification with inhibitors [11]. In the present study, we found that VACV entry into S2 cells, measured by Luc activity, was enhanced less than 2-fold by brief exposure of attached virions to pH 5 buffer compared to 8-fold enhancement of entry into HeLa S3 cells (Fig. 3A). Bafilomycin A1 inhibited VACV entry into BS-C-1 cells by about 50% and exposure of bound virions to pH 5 buffer in the presence of inhibitor allowed entry to recover to 70% of control (Fig. 3C). Remarkably, bafilomycin A1 inhibited entry of VACV in S2 cells by 99% and was still 95% below the control after exposure to pH 5 buffer (Fig. 3C), suggesting that entry occurred predominantly or exclusively through a low pH endosomal route.

We previously showed strain differences in the mode of entry of VACV into mammalian cells. Entry as measured by Luc activity was higher for IHD-J than WR at neutral pH; however, entry of IHD-J was neither accelerated by brief low pH treatment nor inhibited by bafilomycin A1 [12]. In S2 cells, however, the situation was reversed: WR entry was higher than IHD-J (Fig. 3B). The defect was not due to poor binding of the IHD-J strain to S2 cells as determined by flow cytometry using recombinant viruses with GFP fused to a core protein (data not shown). The results with IHD-J were consistent with an inability of S2 cells to support neutral pH entry of VACV.

Viruses enter cells by a variety of endocytic mechanisms [31]. Studies of VACV entry in mammalian cells indicated a requirement for cell signaling and actin dynamics, consistent with macropinocytosis [32] or dynamin 2-dependent fluid phase uptake pathways [33]. VACV infection of S2 cells is reduced to varying degrees by latrunculin A, wortmannin, rottlerin, 5-(N-ethyl-N-isopropyl)amirolide, known inhibitors of actin dynamics or macropinocytosis [6]. We found that genistein, a broad-spectrum tyrosine kinase inhibitor (Fig. 3D), dynasore, a dynamin GTPase inhibitor (Fig. 3E) and blebbistatin (Fig. 3G), a myosin II inhibitor reduced VACV WR entry into both BS-C-1 and S2 cells to similar extents. Cytochalasin D (Fig. 3F), an inhibitor of actin polymerization, reduced entry into BS-C-1 cells by greater than 95%, but inhibited entry into S2 cells by ∼50%. Except for the latter quantitative difference, the entry requirements for VACV WR in S2 and mammalian cells appeared to be similar, with the major difference being the apparent inability to enter S2 cells by an alternative neutral pH route.

### Visualization of VACV infection of S2 cells

**Figure 4:**
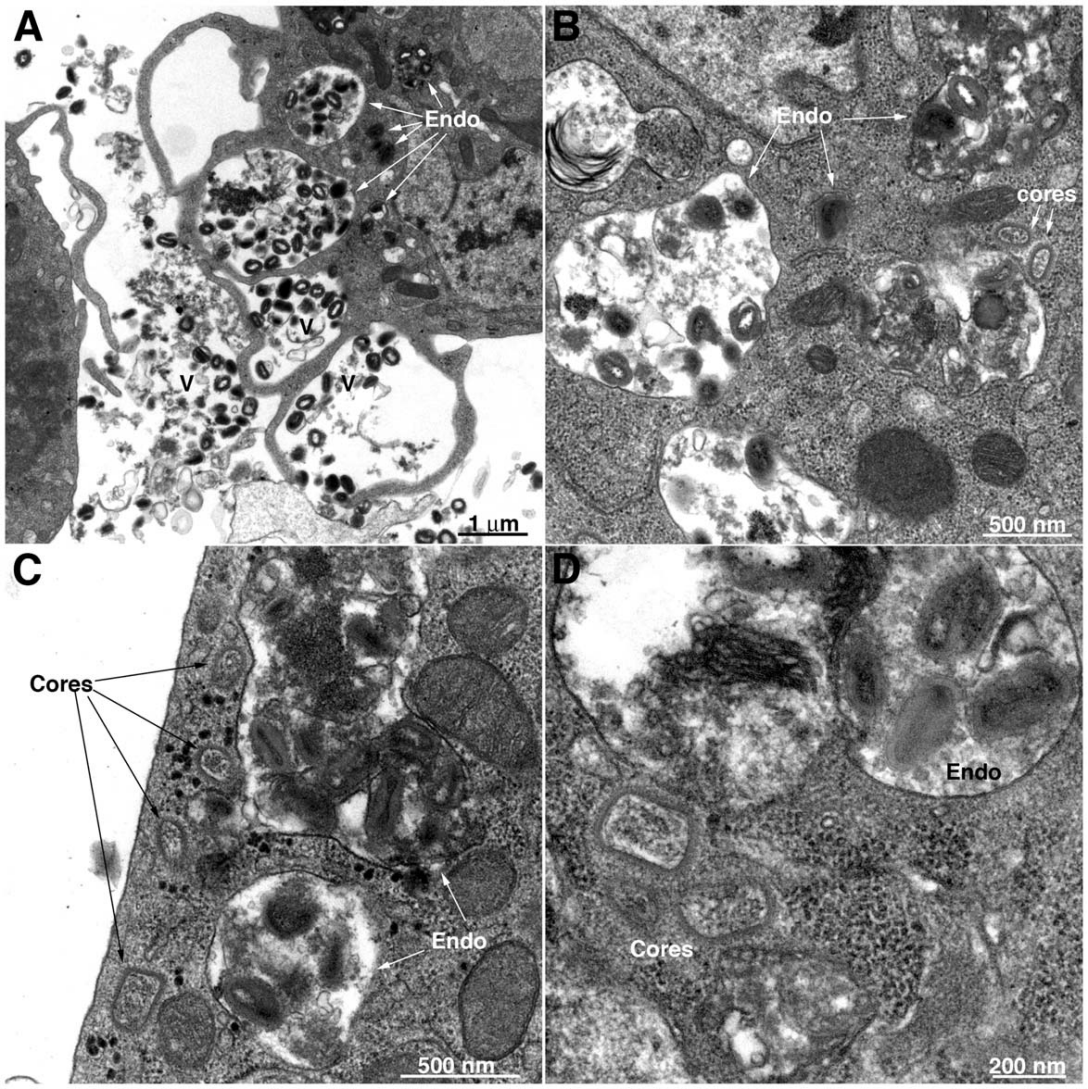
Transmission electron microscopy of VACV infected S2 cells. Purified VACV virions were spinoculated onto cells for 1 h at 4°C at a MOI of 150 PFU per cell. The cells were then incubated at 31°C. **A**) Low magnification of virions associated with cells and in endosomes at 1 h after infection. **B–D**) Higher magnifications showing virions in vesicles and cores in the cytoplasm at 1 h after infection. V, virions; Endo, endosome. A size marker is present at the lower right corner of each panel.

**Figure 5:**
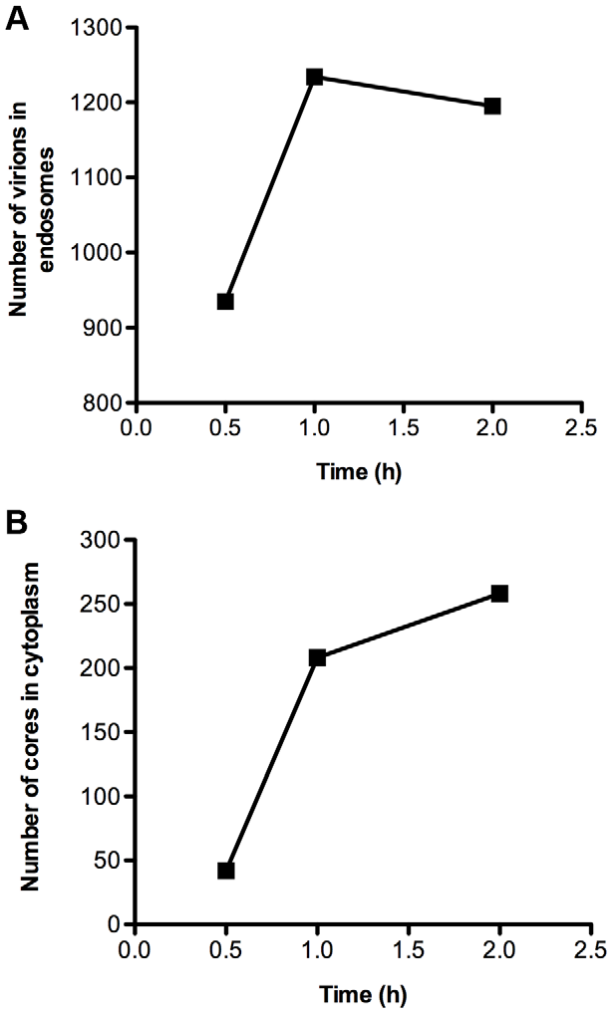
Quantification of virions in endosomes and cores in cytoplasm. Data are from the same experiment used to obtain images in Fig. 4. The numbers of virus particles were counted in single sections of 90 different cells and the totals plotted: **A**) MVs in vesicles; **B**) cores in cytoplasm.

Since the reporter gene assay measures a post-entry event, transmission electron microscopy was employed to visualize VACV infection of S2 cells. After 1 h of incubation at 31°C, numerous virions were at the cell surface particularly near protrusions but none were seen fusing with the plasma membrane (Fig. 4A), in contrast to the situation with BS-C-1 cells [11]. The S2 cells contained large numbers of virions in vesicles whereas cores, with a distinctive oval shape and brush-like surface, were detected in the nearby cytoplasm (Fig. 4B–D). The number of virions in endosomes increased from 0.5 to 1 h whereas the number of cores in the cytoplasm continued to increase for the 2 h period analyzed (Fig. 5A,B). At the latter time, the number of cores was about one-fourth the number of endosomal virions, indicating relatively efficient entry into the cytoplasm.

### Transcriptome analysis of VACV infected S2 cells

**Figure 6:**
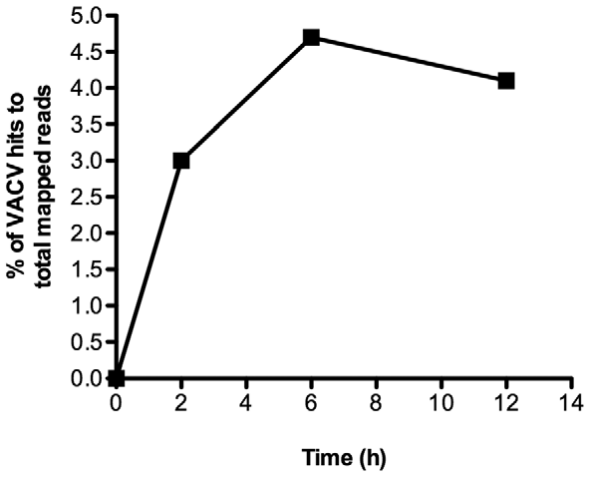
Relative amount of VACV mRNA in infected S2 cells. Total polyadenylated RNA was extracted at various times after infection and subjected to deep RNA sequencing. The sequences were divided into those that mapped to the *Drosophila* and VACV genomes. The percentage of the sequence hits to VACV genome relative to the total mapped reads at progressive time after infection is shown.

The efficient entry of cores into the cytoplasm of S2 cells contrasted with the low Luc activity, suggesting that transcription or translation was limiting. Recently, we showed that the entire VACV transcriptome could be analyzed by deep sequencing of total polyadenylated RNA from HeLa cells [26]. The same technique was applied to infected S2 cells. Total polyadenylated RNA was isolated at 0, 2, 6 and 12 h post infection and cDNAs were prepared and sequenced with an Applied Biosystems SOLiD analyzer. Approximately 7 million to 20 million mappable reads were obtained for each time point and were divided into those that contained cellular or viral sequences. Viral sequence reads represented about 5% of the total at 6 h (Fig. 6), which is in the range (4 to 11%) occurring in HeLa cells prior to DNA replication [26].

**Figure 7:**
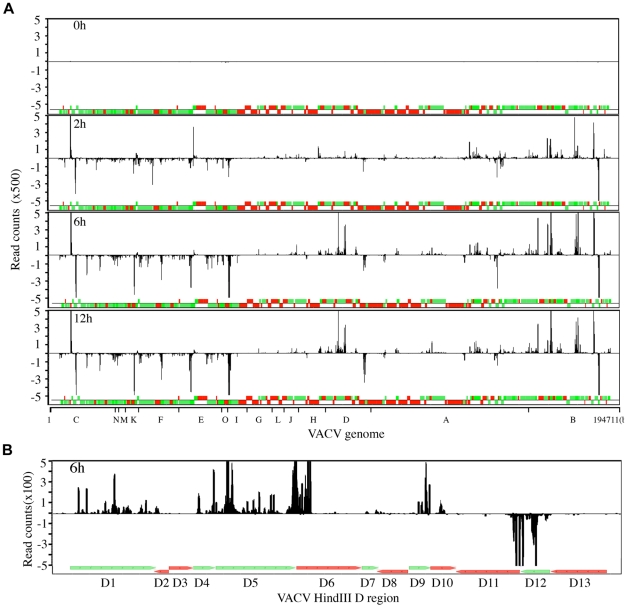
VACV genome-wide transcriptome maps of S2 cells. **A**) The number of viral sequence reads per nucleotide was determined as indicated in the legend to Fig. 6 and displayed over the entire VACV genome with early and post-transcriptional ORFs in green and red, respectively. The counts above the line map to the upper (rightward) strand and counts below the line map to the lower (leftward) strand of the VACV genome. The highest read counts are off-scale in the 2- to 12-h samples for display purposes. The counts were normalized by the total reads of the samples and those duplicated because of their location within the inverted terminal repetition were divided by 2. The HindIII restriction map of the VACV genome is shown at the bottom for reference purposes. **B**) The HindIII D region from the 6 h time point in panel A has been enlarged.

Individual reads were aligned to the VACV genome to construct single-base-resolution maps of the VACV transcriptome in S2 cells. The reads per nucleotide were plotted along the annotated genome, which was color coded for open reading frames (ORFs) expressed before (green) and after (red) DNA replication (Fig. 7A). Reads above the line represent cDNAs prepared from mRNAs transcribed from the upper DNA strand in the rightward direction of the genome, and reads below the line represent mRNAs transcribed from the bottom DNA strand in the leftward direction of the genome. Most of the VACV transcripts detected at 2 h after infection were increased at 6 and 12 h, with the majority mapping to the plus strand at the right end of the genome and the minus strand at the left end of the genome, corresponding to the positions of the majority of early genes. This pattern was very similar to the VACV early transcriptome maps at 0.5 to 2 h in human cells and in the presence of DNA and protein synthesis inhibitors [26]. At 4 h in human cells, however, the transcription pattern changes drastically correlating with the onset of DNA replication followed by late mRNA synthesis [26], whereas this late pattern was not observed even 12 h after infection of S2 cells.

An enlarged view of the HindIII D segment of the VACV genome, indicating the ORFs expressed at early and late times in mammalian cells, is shown in Fig. 7B. The transcripts from VACV-infected S2 cells aligned with the early ORFs but not the late ones (Fig. 7B), precisely as had been found by deep RNA sequencing of RNA from infected HeLa cells [26] and determined by conventional methods [34]. As in HeLa cells, the 3′ ends of some early transcripts overlapped the start of adjacent late transcripts. There was also a general correspondence between the relative numbers of reads for individual ORFs, e.g. the reads for D7 were lowest in both S2 and HeLa cells. The read counts of the VACV ORFs at each time point are listed in Table S1 and illustrate the early expression pattern for the entire genome.

### Protein synthesis in VACV-infected S2 cells

**Figure 8:**
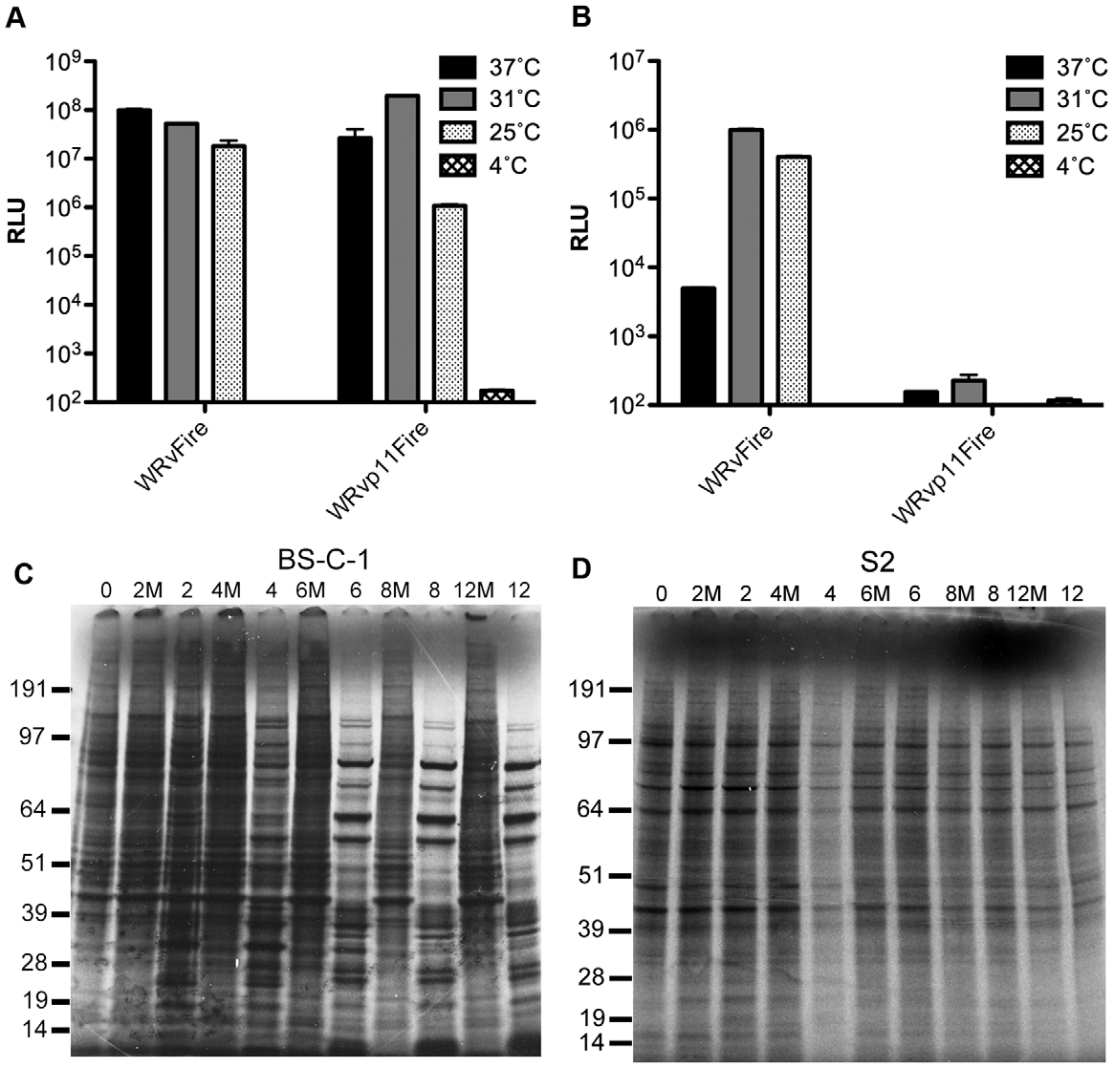
Late gene expression. BS-C-1 (**A**) and S2 (**B**) cells were infected at an MOI of 1 PFU per cell with a recombinant VACV with Luc regulated under the VACV early/late synthetic promoter (WRvFire) or the p11 late promoter (WRvp11Fire) at indicated temperatures. Luc activity was measured at 16 h after infection. Standard error bars were plotted in both panels but are too close to discern in some places. BS-C-1 (**C**) and S2 (**D**) cells were infected with VACV at an MOI of 20 PFU per cell and pulse-labeled with ^35^S-labeled amino acids for 30 min intervals at the times indicated and analyzed by sodium dodecyl sulfate-polyacrylamide gel electrophoresis and autoradiography. The masses of marker proteins in kDA are indicated on the left. M denotes mock-infected cells.

BS-C-1 and S2 cells were infected with recombinant VACVs containing the Luc gene regulated by a synthetic early/late promoter or the p11 late promoter and incubated at 4°C, 25°C, 31°C and 37°C for 16 h. In BS-C-1 cells, the highest level of expression from the early/late and late promoters were at 37°C and 31°C, respectively (Fig. 8A). In S2 cells (Fig. 8B), Luc expression under the early/late promoter was higher at 31°C and 25°C compared to 37°C and the activity was barely above background with the late promoter (note the log scale). These results suggested that VACV is unable to mediate late gene expression in S2 cells.

Pulse-labeling experiments were carried out to confirm the above interpretation and to assess the effects of VACV on expression of cellular proteins. In mammalian cells, host protein synthesis is drastically reduced at 6 h after infection, allowing detection of abundant late protein bands as shown in Fig. 8C. In contrast, VACV had no discernable effect on synthesis of *Drosophila* proteins and intense viral protein bands were not detected at the multiplicity of 20 PFU per cell used (Fig. 8D).

The defect in viral late protein synthesis was confirmed by Western blotting using antibody to A3 core protein (not shown) and by the absence of immature virus particles in thin sections of cells viewed by transmission electron microscopy (not shown). The only sign of de novo gene expression was the clearing of areas of the cytoplasm suggesting pre-factories.

### Expression of intermediate and late proteins from a transfected DNA template

**Figure 9:**
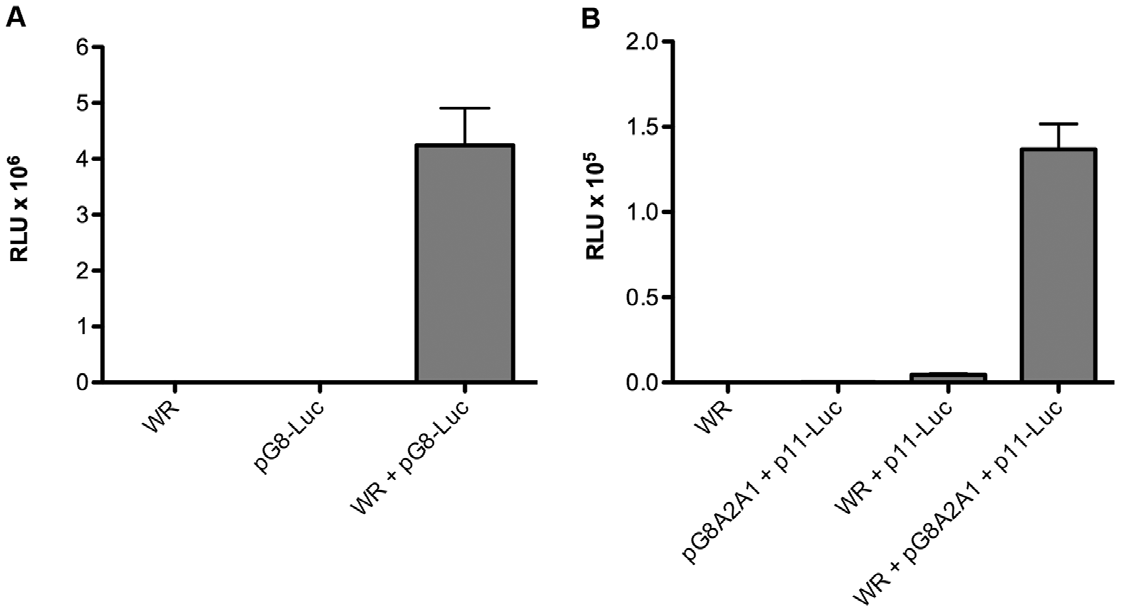
Expression of reporter genes regulated by intermediate and late promoters in transfected plasmids. **A**) Intermediate expression. S2 cells were mock infected or infected with VACV strain WR at a MOI of 1 PFU per cell and mock transfected or transfected with a plasmid containing the Luc ORF regulated by the G8R intermediate promoter. Luc activity was measured at 16 h. **B**) S2 cells were infected with VACV strain WR and transfected with a plasmid containing the Luc ORF regulated by the late p11 promoter and cotransfected or not with a second plasmid containing the three late transcription factor genes (G8R, A1L, A2L) regulated by intermediate promoters. Luc activity was measured at 16 h. Standard error bars were plotted in both panels but are too close to discern in some places.

Based on the results so far, the abortive VACV replication in S2 cells could be due to a primary block at the stage of viral DNA replication or intermediate and late transcription. Except for the ancillary use of cellular DNA ligase 1 [35], no mammalian host factors have yet been found for VACV DNA replication, whereas in vitro studies have demonstrated specific host factor requirements for both intermediate and late transcription [36], [37]. The DNA replication requirement for intermediate gene expression in mammalian cells can be overcome by DNA transfection [38], since the viral transcription factors needed are early gene products [39]. We found that Luc expression occurred in VACV-infected S2 cells when a plasmid containing the Luc gene under an intermediate promoter was transfected (Fig. 9A), indicating the synthesis of functional viral RNA polymerase and intermediate transcription factors. Control experiments indicated no expression when either the virus or expression plasmid was omitted (Fig. 9A).

The DNA replication requirement for late gene expression in mammalian cells can be bypassed by transfecting plasmids containing the three late transcription factor genes, which have intermediate promoters, allowing detection of a reporter gene with a late promoter in VACV-infected cells [40]. This was accomplished in VACV-infected S2 cells by measuring expression of Luc regulated by the late p11 promoter (Fig. 9B). Note that Luc expression depended on VACV infection and co-transfection of the intermediate transcription factor plasmid. These results indicated that both intermediate and late transcription and translation could occur in S2 cells provided DNA templates are transfected.

### DNA replication

**Figure 10:**
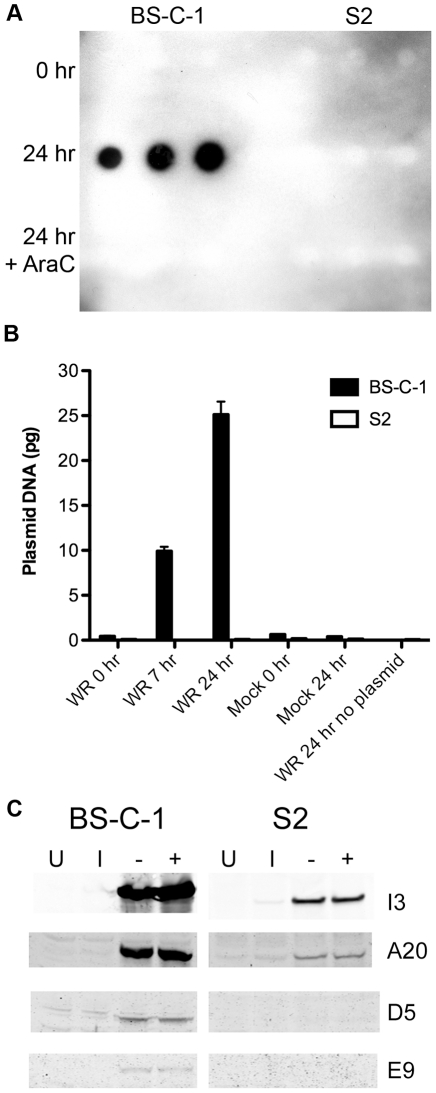
DNA replication. **A**) VACV genome replication. BS-C-1 and S2 cells were infected with VACV strain WR (MOI of 5 PFU/cell) in the presence or absence of AraC. At 0 and 24 h, the amount of VACV DNA was determined in triplicate by slot blot hybridization using digoxigenin-dUTP labeled F17R probe. **B**) Plasmid replication. BS-C-1 and S2 cells were infected with VACV strain WR (MOI of 3 PFU/cell) and then transfected with a plasmid. At 0, 7 and 24 h, plasmid sequences were quantified by real-time PCR. Mock infected cells and incubations without plasmids were used as controls. Standard error bars were plotted but are too close to discern in some places. **C**) Western blotting of VACV DNA replication proteins. BS-C-1 and S2 cells were infected with VACV (MOI of 20 PFU/cell), incubated after overnight at 31°C in the presence (+) and absence (-) of AraC analyzed by SDS-polyacrylamide gel electrophoresis and Western blotting with antibody to the I3, A20, D5 and E9 proteins. Uninfected cells (U) and cells harvested after inoculation (I) were used as controls.

The results obtained so far suggested a block in VACV DNA replication in S2 cells. This defect was verified by Southern blotting: viral DNA was detected in BS-C-1 cells but not in S2 cells (Fig. 10A). The absence of viral DNA synthesis could be due to inaccessibility of the packaged viral genome, a failure of protein uncoating or to a specific replication defect. To assess the latter, a naked plasmid was transfected into BS-C-1 and S2 cells, since circular DNAs without specific viral sequences replicate efficiently in mammalian cells infected with VACV [41], [42]. Moreover, plasmid replication is dependent on expression of each of the viral genes known to be required for genome replication [43]. Plasmid replication, determined by real time PCR, increased over time in VACV infected BS-C-1 cells but not in infected S2 cells or the mock infected or transfected controls (Fig 10B). The inability of the plasmid to replicate in infected S2 cells indicated a specific block in DNA synthesis.

The defect in plasmid replication could be due to a host restriction or insufficient amounts of viral replication proteins. We evaluated the latter by Western blotting with available antibodies to viral replication proteins. The proteins I3 and A20 were detected in BS-C-1 and in lesser amounts in S2 cells, though equal amounts of protein lysates were loaded in the gels (Fig. 10C). D5 and E9 proteins from BS-C-1 cells were detected as faint bands but were below the level of detection in S2 cells (Fig. 10C). Since the mRNA levels for all four proteins were comparable in S2 and BS-C-1 cells (Table S1, ref. 58), inefficient translation could contribute to the defect in DNA replication. The relatively weak staining of bands even in BS-C-1 cells, however, made it difficult to interpret the significance of the lower staining in S2 cells.

## Discussion

*Drosophila* S2 cells are used for high-level heterologous protein synthesis employing DNA or baculovirus expression systems [44] and for genome-wide siRNA knockdown [30]. Insect-specific RNA viruses as well as several human RNA viruses, including vesicular stomatitis virus, Sindbis virus, Rift Valley fever virus, Dengue virus, and West Nile virus can productively infect S2 cells [cited in [30]. During a recent RNAi kinome screen, Moser and co-workers [6] found that VACV abortively infected S2 cells and cited unpublished data that DNA replication was blocked. We confirmed the abortive replication of VACV in *Drosophila* S2 cells and analyzed each step in the replication cycle to better understand the host restriction and provide the basis for comprehensive genome-wide siRNA screens.

As S2 cells are derived from phagocytic haemocytes, it was important to establish that entry occurred via the VACV fusion machinery and not by another mechanism. We demonstrated that entry was inhibited by a MAb to L1 and was dependent on the presence of A28, two of the proteins known to be essential for VACV entry in mammalian cells. VACV entry into mammalian cells occurs at the plasma membrane and via low pH-dependent endocytosis simultaneously. It was surprising, therefore, to find that entry of VACV strain WR into S2 cells was inhibited 99% by bafilomycin A1, an inhibitor of endosomal acidification, and could not be enhanced by acidification of the medium, suggesting that neutral pH fusion with the plasma membrane did not occur to a significant extent. Indeed, numerous intact virus particles in vesicles and free cores in the cytoplasm were seen by transmission electron microscopy, but there were no images of plasma membrane fusion events, which are readily detected with mammalian cells. Consistent with these results, we found that S2 cells were less permissive for the IHD-J strain of VACV, which enters mammalian cells primarily through a neutral pH mechanism [12], [45], than the WR strain. Specific entry receptors have not been defined for VACV although the presence of glycosaminoglycans and cholesterol on the cell surface are important for entry of VACV into mammalian cells [46], [47]. S2 cells contain cell surface heparan sulfate proteoglycans [48] but are auxotrophic for cholesterol. However, S2 cells were grown with cholesterol containing serum and cholesterol supplementation did not enhance entry as measured by Luc expression (ZB, unpublished). Nevertheless, other differences in the distribution of lipids in mammalian and insect cells could be responsible for the inability of VACV to fuse with the plasma membrane. The apparent use of a single entry pathway in S2 cells could simplify the identification of host factors required for this step by RNAi methods.

The presence of RNA polymerase and transcription factors in the core of infectious VACV virions allows transcription of early genes to occur in the cytoplasm soon after entry. Deep RNA sequencing of HeLa cells infected with VACV defined 118 early ORFs that were expressed between 30 and 120 min and 93 additional ORFs that were expressed at 4 h, after viral DNA replication [26]. Using a similar approach, we demonstrated expression of the same set of early genes but no post-replication genes even when analyzed 12 h after infection. Moreover, the VACV early transcripts comprised a similar percentage of the total polyadenylated RNA in S2 and HeLa cells, indicating efficient entry and early transcription. The inability to detect late protein synthesis by pulse-labeling infected cells was consistent with the absence of late mRNAs.

Transcription of intermediate and late genes occurs in cytoplasmic viral factories and depends on de novo synthesis of a large number of early proteins including a multi-subunit DNA-dependent RNA polymerase and stage-specific transcription factors in addition to a DNA template. Evidence for additional host factors has been obtained by in vitro complementation studies. Therefore, the failure to transcribe intermediate and late genes could have multiple causes. However, by transfecting plasmid templates, we bypassed the requirement for replicated viral genomic DNA and succeeded in demonstrating intermediate and late gene expression. Thus, the early proteins needed for intermediate gene expression were synthesized and the data strongly suggested a block in viral genome replication in S2 cells. Indeed, we could not detect replication of the viral genome. This defect could be due to a failure to completely uncoat the packaged DNA, failure to synthesize adequate amounts of viral replication proteins, absence of a required mammalian host factor, or presence of an inhibitory host factor. Attempts to determine the presence of an uncoating defect as a decrease in viral cores by confocal microscopy or an increase in naked DNA by real time PCR were not sufficiently quantitative in S2 cells (ZB, unpublished). However, the capability of VACV-infected S2 cells to replicate exogenous DNA, which is dependent on all known viral replication proteins [43], can be tested by transfection of plasmids. Our inability to detect plasmid synthesis indicated that there was a primary block in DNA replication, though it did not eliminate an additional block in genome uncoating. One possibility, consistent with Western blotting, is that the lower amounts of early proteins were not sufficient to catalyze DNA replication, an explanation provided for results obtained in mammalian cells with a conditional lethal mutant in the VACV capping enzyme [49]. However, an even larger number of early proteins are needed for intermediate transcription than for DNA replication and intermediate expression could be readily demonstrated by transfection of a DNA template. An interesting possibility is that cytoplasmic DNA replication is blocked as a host defense mechanism in S2 cells. The ability of the DNA binding protein Barrier to Autointegration Factor (BAF) to inhibit replication of VACV B1 kinase mutants provides a model for such an activity [50]. The question of whether a host inhibitory factor is present or a host stimulatory factor is missing might be answered by carrying out a comprehensive siRNA screen to test the former and a forward genetic approach by transfecting a human cDNA library into S2 cells to test the latter, in each case using plasmid replication or intermediate gene expression as the read out.

## Materials and Methods

### Cells and viruses

Mammalian BS-C-1 (CCL-26) and HeLa S1 (CCL-2.2) cells were obtained from the American Type Culture Collection (Manassas, VA) and maintained in Earle's minimum essential medium (EMEM) supplemented with 10% fetal bovine serum, 100 units/ml of penicillin, 100 µg of streptomycin per ml (Quality Biologicals, Gaithersburg, MD) and 2 mM L-glutamine. S2 cells were obtained from the *Drosophila* Genomics Resource Center and maintained in Schneider Medium (Invitrogen, Carlsbad, CA) supplemented with 10% heat inactivated fetal bovine serum. The Western Reserve (WR) strain of VACV (ATTC VR-1354; GenBank accession number NC_006998), WRvFire [11] and IHD-JvFire [12] were propagated and the MV purified by sucrose gradient sedimentation as previously described [12], [51]. Recombinant WR virus with the firefly luciferase (Luc) open reading frame attached to the P11 late promoter and inserted between the F12 and F13 open reading frames was generated as previously described [12] and named WRvp11Fire. The inserted DNA of the purified recombinant virus was verified by sequencing. Similarly, vA28-HAi-Fire was created by inserting the firefly Luc open reading frame with the synthetic early/late promoter between the F12 and F13 open reading frames of vA28-HAi [52], a WR strain virus with an IPTG-regulated HA-tagged A28 protein.

### Luc assay

Cells were seeded onto 24-well plates and incubated overnight at 37°C for mammalian cells and 25°C for S2 cells. Cells were chilled for 10 min at 4°C and infected with purified MVs at a multiplicity of infection (MOI) of 1–2 plaque forming units (PFU) per cell. The virions were allowed to adsorb to cells for 1 h at 4°C in EMEM-2.5 (EMEM supplemented with 2.5% fetal bovine serum, 2 mM L-glutamine, 100 units/ml penicillin and 100 µg/ml streptomycin). Unattached virus was removed by washing and the infection was allowed to proceed at 31°C unless indicated otherwise in the Results section. Cells were harvested by incubation with 200 µl of Cell Culture Lysis Reagent (Promega, Madison, WI) for 30 min at room temperature on an orbital shaker. The Luc assay was performed by adding 20 µl of cell lysate to 100 µl of Luc activity assay substrate (Promega), mixed, and chemiluminescence measured using a luminometer (Berthold Sirius, Bad Wilbad, Germany).

### Electron microscopy

Purified MVs (150 PFU/cell) were suspended in 0.75 ml EMEM-2.5 and added to S2 cells that were plated on 6-well dishes and pre-chilled to 4°C. Plates were wrapped in polyvinylidene chloride (Saran Wrap) and placed in a 75006449 C bucket in a Heraeus rotor for a Legend RT Sorvall centrifuge. Virus was spinoculated [53] onto cells at 650×*g* for 1 h at 4°C. The cells were gently washed and incubated at 31°C for 30, 60 or 120 min, fixed in 2% glutaraldehyde/0.1 M sodium cacodylate buffer, washed in 0.1 M sodium cacodylate buffer, post-fixed with reduced osmium tetroxide, and washed in buffer. Cells were dehydrated successively in 50%, 70%, and 100% ethanol and then propylene oxide. The samples were embedded in EMbed 812 (Electron Microscopy Sciences, Hatfield, PA) and sections were cut on a Leica EM UC7 ultramicrotome (Leica Microsystems, Wetzlar, Germany). Thin sections were stained with 7% uranyl acetate in 50% ethanol and then 0.01% lead citrate. Sections were reviewed and photographed on the FEI Tecnai G2 Spirit transmission electron microscope fitted with a Gatan CCD camera (FEI Company, Hillsboro, OR). Chemicals were purchased from Electron Microscopy Sciences.

### Deep sequencing of RNA from VACV- infected S2 cells

S2 cells were infected with purified VACV at a MOI of 20 PFU per cell. Preparation and sequencing was carried out as described [26] with minor modifications. Briefly, polyadenylated RNA was isolated from VACV-infected S2 cells by two rounds of selection using the Dynabeads mRNA Direct Kit (Invitrogen). The strand-specific cDNA library was prepared with the Whole Transcriptome Analysis Kit (Ambion, Austin, TX) using adaptor A and sequenced with the Applied Biosystems SOLiD 3 system. Multiple samples were sequenced together using barcodes. The raw reads were processed with the Applied Biosystems Whole Transcriptome Analysis (WTA) Pipeline and split-read mapper tool and mapped to VACV genome (NC_006998) with 2 mismatches allowed. VACV transcriptome maps were displayed and visualized with Mochiview [54]. The cDNA sequences were deposited in the Sequence Read Archive of the National Library of Medicine under submission access numbers SRA27288 and SRP004868.

### DNA replication

BS-C-1 and S2 cells were infected with VACV in 24-well dishes at 31°C for 24 h. Cells were lysed in 10 X SSC (1.5 M NaCl and 0.15 M sodium citrate pH 7.0) containing 1 M ammonium acetate by three freeze-thaw cycles. Lysates were cleared by incubation with Proteinase K (Sigma Aldrich, St. Louis, MO) followed by sonication. DNA was denatured with 0.4 M NaOH and 10 mM EDTA for 10 min at 100°C and spotted on Hybond-N+ (Amersham, Piscataway, NJ) nylon membrane under vacuum. The blot was washed with 10 X SSC, denatured with 0.5 M NaOH, 1.5 M NaCl and neutralized with 1.5 M NaCl, 1 M Tris base before hybridization using QuiK-Hyb solution (Stratagene, Santa Clara, CA). VACV DNA was detected using digoxigenin-dUTP labelled F17R gene probe (DIG High Prime DNA labeling and detection kit, Roche, Indianapolis, IN). Analysis of plasmid replication was carried out by real-time PCR as previously described [35], [43]. Briefly, 0.1 µg of p716 plasmid [55] and 3.9 µg of salmon sperm carrier DNA were mixed with 10 µg of lipofectamine 2000 (Invitrogen) and uninfected cells were transfected according to the manufacturer's instructions. After 24 h, the cells were infected with VACV strain WR at a multiplicity of 3 PFU per cell. Cells were then washed twice with Opti-MEM (Invitrogen) and incubated for various times, harvested and the DNA isolated using the QIAamp DNA Blood Kit (Qiagen) according to the manufacturer's instructions. DNA was digested with restriction enzyme *Dpn*I (New England Biolabs, Ipswich, MA). Oligonucleotides P1 (5′CAACTAAATGTGCAAGCAATGTAATTC3′) and P2 (5′CATCCTGCCCCTTGCTGT3′) were designed with Primer Express software supplied by Applied Biosystems. Reactions were carried out using SYBR Green PCR master mix (Applied Biosystems), 10 µM of each primer, and 1 ng of DNA in a total volume of 50 µl in an RealPlex sequence detection system and software (Eppendorf, Westbury, NY). DNA was amplified with 40 cycles at 95°C for 15 sec and 60°C for 60 sec.

### Western blotting

Protein concentrations of cell-lysates were determined using the micro bicinchonic acid kit (Pierce, Rockford, IL). Equal amounts of cell-lysates (100 µg) were resolved by sodium dodecyl sulfate polyacrylamide gel electrophoresis on 4%–12% Novex NuPAGE acrylamide gels with 2-(*N*-morpholino)ethansulfonic-sodium dodecyl sulfate running buffer and transferred to nitrocellulose membranes using mini iBlot gel transfer stacks (Invitrogen). After transfer, the membranes were blocked with 5% nonfat milk in phosphate buffered saline (PBS) containing 0.05% Tween-20 (PBS-T). Membranes were then incubated with a monoclonal antibody (MAb) against the I3 protein [56] or polyclonal sera against VACV DNA polymerase E9 [57], A20 protein [58], or D5 protein [59] overnight at 4°C and washed with PBS-T and PBS. The membrane was then incubated with donkey anti-mouse IRDye 680/800 or donkey anti-rabbit IRDye 680/800 for 2 h at room temperature, washed with PBS-T and PBS and developed using a LI-COR Odyssey infrared imager (LI-COR Biosciences, Lincoln, NE).

## Supporting Information

Table S1 **Normalized read counts of VACV ORFs in infected** ***Drosophila*** **S2 cells.**
(DOC)
