# Supplementary material for: Drosophila S2 Cells Are Non-Permissive for Vaccinia Virus DNA Replication Following Entry via Low pH-Dependent Endocytosis and Early Transcription
Source: PLoS One. 2011 Feb 15;6(2):e17248. doi: 10.1371/journal.pone.0017248 (PMC3039670; doi:10.1371/journal.pone.0017248)
Supplement: Table S1 — Normalized read counts of VACV ORFs in infected Drosophila S2 cells. (DOC) [file pone.0017248.s001.doc]

Table S1. Normalized read counts of VACV ORFs in infected *Drosophila* S2 cells^a,b^

| VACV-WR | VACV-COP | Temporal expression class^c^ | 0 h | 2 h | 6 h | 12 h |
| --- | --- | --- | --- | --- | --- | --- |
| VACWR001/218 | C23L | E1.1 | 0 | 343 | 426 | 800 |
| VACWR002/217 | pseudogene | E1.2 | 0 | 3 | 32 | 52 |
| VACWR003/216 | no ortholog | PR | 0 | 0 | 0 | 0 |
| VACWR004/215 | C22L | E1.2 | 0 | 649 | 242 | 95 |
| VACWR005/214 | pseudogene | E1.2 | 0 | 307 | 123 | 159 |
| VACWR006/213 | C21L | E1.2** | 0 | 66 | 39 | -1 |
| VACWR007/212 | no ortholog | E1.2 | 0 | 96 | 45 | 95 |
| VACWR008/211 | C19L | PR | 0 | 50 | 36 | 35 |
| VACWR009/210 | C11R | E1.1 | 0 | 38500 | 59971 | 44262 |
| VACWR010/209 | C10L | E1.2 | 0 | 336 | 340 | 323 |
| VACWR011/208 | no ortholog | E1.2 | 0 | 293 | 186 | 221 |
| VACWR012/207 | no ortholog | E1.2 | 0 | 11 | 114 | 121 |
| VACWR013 | no ortholog | E1.2 | 0 | 566 | 231 | 195 |
| VACWR014 | no ortholog | E1.2 | 0 | 24 | 56 | 61 |
| VACWR015 | no ortholog | E1.2 | 0 | 43 | 90 | 0 |
| VACWR016 | no ortholog | E1.2** | 0 | 0 | 11 | 125 |
| VACWR017 | no ortholog | E1.2 | 0 | -9 | 661 | 612 |
| VACWR018 | no ortholog | E1.1 | 0 | 2314 | 9431 | 7966 |
| VACWR019 | C9L | E1.2 | 0 | 202 | 454 | 369 |
| VACWR020 | C8L | PR | 0 | 36 | 4 | 16 |
| VACWR021 | C7L | E1.2 | 0 | 308 | 46 | 67 |
| VACWR022 | C6L | E1.1 | 0 | 978 | 346 | 248 |
| VACWR023 | C5L | E1.2 | 0 | 536 | 2618 | 2732 |
| VACWR024 | C4L | E1.2 | 0 | 337 | 309 | 346 |
| VACWR025 | C3L | PR | 0 | 64 | 42 | 41 |
| VACWR026 | C2L | E1.2 | 0 | 272 | 290 | 255 |
| VACWR027 | C1L | E1.1 | 0 | 822 | 1040 | 1223 |
| VACWR028 | N1L | E1.2 | 0 | 308 | 1417 | 1755 |
| VACWR029 | N2L | E1.1 | 0 | 1662 | 2220 | 2698 |
| VACWR030 | M1L | E1.2 | 0 | 445 | 1499 | 1797 |
| VACWR031 | M2L | E1.2 | 0 | 516 | 603 | 602 |
| VACWR032 | K1L | E1.1 | 0 | 718 | 162 | 152 |
| VACWR033 | K2L | PR | 0 | 30 | 76 | 48 |
| VACWR034 | K3L | E1.1 | 0 | 992 | 834 | 478 |
| VACWR035 | K4L | PR* | 0 | 247 | 208 | 248 |
| VACWR036 | pseudogene | E1.1** | 0 | 3334 | 18469 | 19142 |
| VACWR037 | K5L | E1.1 | 0 | 1201 | 795 | 674 |
| VACWR038 | K6L | E1.1 | 0 | 465 | 137 | 191 |
| VACWR039 | K7R | E1.1 | 0 | 964 | 839 | 931 |
| VACWR040 | F1L | E1.2 | 0 | 2656 | 2222 | 1903 |
| VACWR041 | F2L | E1.2 | 0 | 1020 | 3819 | 2727 |
| VACWR042 | F3L | E1.2 | 0 | 143 | 427 | 377 |
| VACWR043 | F4L | E1.2 | 0 | 913 | 1973 | 1431 |
| VACWR044 | F5L | E1.2 | 0 | 688 | 494 | 465 |
| VACWR045 | F6L | E1.2 | 0 | 326 | 55 | 112 |
| VACWR046 | F7L | E1.2 | 0 | 7435 | 1077 | 760 |
| VACWR047 | F8L | E1.2 | 0 | 1000 | 538 | 382 |
| VACWR048 | F9L | PR | 0 | 46 | 45 | 20 |
| VACWR049 | F10L | PR* | 0 | 51 | 127 | 247 |
| VACWR050 | F11L | E1.1 | 0 | 1916 | 4987 | 5444 |
| VACWR051 | F12L | E1.2 | 0 | 259 | 207 | 118 |
| VACWR052 | F13L | PR | 0 | 44 | 45 | 41 |
| VACWR053 | F14L | E1.1 | 0 | 1502 | 1562 | 1060 |
| VACWR053.5 | F14.5L | E1.2** | 0 | 174 | 75 | 115 |
| VACWR054 | F15L | E1.1 | 0 | 519 | 988 | 608 |
| VACWR055 | F16L | E1.2 | 0 | 93 | 177 | 191 |
| VACWR056 | F17R | PR | 0 | 85 | 5 | 95 |
| VACWR057 | E1L | E1.2 | 0 | 170 | 592 | 356 |
| VACWR058 | E2L | E1.2 | 0 | 201 | 480 | 265 |
| VACWR059 | E3L | E1.1 | 0 | 3899 | 5704 | 8440 |
| VACWR060 | E4L | E1.1 | 0 | 949 | 847 | 921 |
| VACWR061 | E5R | E1.1 | 0 | 2738 | 698 | 701 |
| VACWR062 | E6R | PR* | 0 | 83 | 61 | 77 |
| VACWR063 | E7R | PR | 0 | 69 | 21 | 70 |
| VACWR064 | E8R | PR | 0 | 50 | -7 | 5 |
| VACWR065 | E9R | E1.2 | 0 | 530 | 1394 | 1208 |
| VACWR066 | E10R | PR | 0 | 115 | 1 | -10 |
| VACWR067 | E11R | PR* | 0 | 403 | 257 | 89 |
| VACWR068 | O1L | E1.1 | 0 | 651 | 910 | 559 |
| VACWR069 | O2L | PR | 0 | 185 | 22 | 85 |
| VACWR069.5 | O3L | PR | 0 | -9 | 22 | -18 |
| VACWR070 | I1L | PR | 0 | 404 | 117 | 153 |
| VACWR071 | I2L | PR* | 0 | 777 | 2472 | 3898 |
| VACWR072 | I3L | E1.1 | 0 | 3527 | 25540 | 23243 |
| VACWR073 | I4L | E1.2 | 0 | 105 | 297 | 144 |
| VACWR074 | I5L | PR | 0 | 53 | 81 | 30 |
| VACWR075 | I6L | PR | 0 | 21 | 66 | 32 |
| VACWR076 | I7L | PR | 0 | 17 | 7 | 2 |
| VACWR077 | I8R | PR | 0 | 40 | 17 | 27 |
| VACWR078 | G1L | PR | 0 | 30 | 3 | 0 |
| VACWR079 | G3L | PR | 0 | 24 | 0 | 0 |
| VACWR080 | G2R | E1.2 | 0 | 184 | 586 | 488 |
| VACWR081 | G4L | PR | 0 | 3 | 0 | 3 |
| VACWR082 | G5R | E1.2 | 0 | 70 | 59 | 100 |
| VACWR083 | G5.5R | E1.2 | 0 | 1149 | 473 | 419 |
| VACWR084 | G6R | PR* | 0 | 97 | 23 | 16 |
| VACWR085 | G7L | PR | 0 | 67 | -1 | 11 |
| VACWR086 | G8R | PR | 0 | 0 | 0 | 1 |
| VACWR087 | G9R | PR | 0 | 11 | 0 | 3 |
| VACWR088 | L1R | PR | 0 | 122 | 6 | 5 |
| VACWR089 | L2R | E1.1 | 0 | 1345 | 617 | 879 |
| VACWR090 | L3R | PR | 0 | 35 | 10 | 11 |
| VACWR091 | L4R | PR | 0 | 40 | 14 | 1 |
| VACWR092 | L5R | PR | 0 | 3 | 18 | 10 |
| VACWR093 | J1R | PR | 0 | 0 | 7 | 0 |
| VACWR094 | J2R | E1.2 | 0 | 135 | 53 | 63 |
| VACWR095 | J3R | E1.2 | 0 | 241 | 251 | 158 |
| VACWR096 | J4R | E1.2 | 0 | 404 | 677 | 735 |
| VACWR097 | J5L | PR | 0 | 7 | 0 | 16 |
| VACWR098 | J6R | E1.2 | 0 | 279 | 586 | 376 |
| VACWR099 | H1L | PR* | 0 | 872 | 773 | 365 |
| VACWR100 | H2R | PR | 0 | 0 | 0 | 30 |
| VACWR101 | H3L | PR | 0 | 48 | 22 | 17 |
| VACWR102 | H4L | PR | 0 | 8 | 2 | 7 |
| VACWR103 | H5R | E1.1 | 0 | 1633 | 1786 | 2857 |
| VACWR104 | H6R | PR* | 0 | 299 | 292 | 489 |
| VACWR105 | H7R | PR | 0 | 50 | 32 | 62 |
| VACWR106 | D1R | E1.2 | 0 | 185 | 857 | 909 |
| VACWR107 | D2R | PR | 0 | 5 | 1 | 16 |
| VACWR108 | D3R | PR | 0 | 55 | 2 | 11 |
| VACWR109 | D4R | E1.2 | 0 | 310 | 1417 | 2761 |
| VACWR110 | D5R | E1.2 | 0 | 457 | 3849 | 4591 |
| VACWR111 | D6R | PR* | 0 | 410 | 1788 | 1656 |
| VACWR112 | D7R | E1.2 | 0 | 590 | 195 | 284 |
| VACWR113 | D8L | PR | 0 | 13 | 5 | 17 |
| VACWR114 | D9R | E1.1 | 0 | 1036 | 1706 | 1165 |
| VACWR115 | D10R | PR* | 0 | 462 | 311 | 245 |
| VACWR116 | D11L | PR* | 0 | 619 | 1066 | 1417 |
| VACWR117 | D12L | E1.2 | 0 | 604 | 1847 | 3276 |
| VACWR118 | D13L | PR | 0 | 55 | 17 | 17 |
| VACWR119 | A1L | PR | 0 | 0 | 9 | 0 |
| VACWR120 | A2L | PR | 0 | 30 | 0 | 1 |
| VACWR121 | A2.5L | PR | 0 | 12 | -1 | -1 |
| VACWR122 | A3L | PR | 0 | 141 | 56 | 62 |
| VACWR123 | A4L | E1.2 | 0 | 397 | 571 | 434 |
| VACWR124 | A5R | E1.2 | 0 | 585 | 526 | 1052 |
| VACWR125 | A6L | PR | 0 | 93 | 10 | 19 |
| VACWR126 | A7L | PR | 0 | 8 | 5 | 8 |
| VACWR127 | A8R | E1.1 | 0 | 451 | 951 | 1040 |
| VACWR128 | A9L | PR | 0 | -5 | -5 | -2 |
| VACWR129 | A10L | PR | 0 | 43 | 2 | 13 |
| VACWR130 | A11R | PR | 0 | 106 | 2 | 24 |
| VACWR131 | A12L | PR | 0 | 2 | 8 | 18 |
| VACWR132 | A13L | PR | 0 | -2 | -2 | 52 |
| VACWR133 | A14L | PR | 0 | 47 | -8 | 29 |
| VACWR134 | A14.5L | PR | 0 | 112 | 0 | 0 |
| VACWR135 | A15L | PR | 0 | 12 | -6 | -6 |
| VACWR136 | A16L | PR | 0 | -1 | 0 | 4 |
| VACWR137 | A17L | PR | 0 | -1 | 13 | 23 |
| VACWR138 | A18R | E1.2 | 0 | 54 | 335 | 229 |
| VACWR139 | A19L | PR | 0 | 0 | 0 | 12 |
| VACWR140 | A21L | PR | 0 | 79 | 0 | 0 |
| VACWR141 | A20R | E1.2 | 0 | 63 | 416 | 507 |
| VACWR142 | A22R | PR* | 0 | 210 | 667 | 956 |
| VACWR143 | A23R | E1.2 | 0 | 59 | 619 | 997 |
| VACWR144 | A24R | E1.2 | 0 | 291 | 339 | 292 |
| VACWR145 | no ortholog | PR | 0 | 0 | 19 | 46 |
| VACWR146 | no ortholog | PR | 0 | 38 | 56 | 18 |
| VACWR147 | no ortholog | PR | 0 | 18 | -6 | -2 |
| VACWR148 | A25L | PR | 0 | -2 | -2 | 2 |
| VACWR149 | A26L | PR | 0 | 32 | 6 | 14 |
| VACWR150 | A27L | PR | 0 | -13 | 16 | 18 |
| VACWR151 | A28L | PR | 0 | 0 | 18 | 6 |
| VACWR152 | A29L | E1.2 | 0 | 311 | 1077 | 1009 |
| VACWR153 | A30L | PR | 0 | 474 | 250 | 207 |
| VACWR153.5 | A30.5L | PR | 0 | 143 | 0 | 19 |
| VACWR154 | A31R | E1.2 | 0 | 783 | 283 | 168 |
| VACWR155 | A32L | PR | 0 | 57 | 7 | 14 |
| VACWR156 | A33R | E1.1 | 0 | 1108 | 677 | 576 |
| VACWR157 | A34R | PR | 0 | 0 | 18 | 20 |
| VACWR158 | A35R | E1.1 | 0 | 1766 | 1450 | 1635 |
| VACWR159 | A36R | E1.1 | 0 | 856 | 1540 | 1583 |
| VACWR160 | A37R | E1.1 | 0 | 1598 | 1890 | 1672 |
| VACWR161 | pseudogene | E1.1 | 0 | 1236 | 631 | 715 |
| VACWR162 | A38L | PR | 0 | 47 | 5 | 8 |
| VACWR163 | A39R | PR* | 0 | 72 | 34 | 95 |
| VACWR164 | A39R | PR | 0 | 35 | 9 | 0 |
| VACWR165 | A40R | E1.2 | 0 | 665 | 2284 | 1603 |
| VACWR166 | A41L | E1.2 | 0 | 284 | 118 | 65 |
| VACWR167 | A42R | PR | 0 | -3 | 9 | -3 |
| VACWR168 | A43R | PR | 0 | 13 | 23 | 39 |
| VACWR169 | 268 | E1.2 | 0 | 534 | 46 | 76 |
| VACWR170 | A44L | E1.2 | 0 | 1707 | 2850 | 1527 |
| VACWR171 | A45R | PR | 0 | 58 | 7 | 21 |
| VACWR172 | A46R | E1.1 | 0 | 1963 | 1330 | 1745 |
| VACWR173 | A47L | E1.2 | 0 | 1845 | 307 | 188 |
| VACWR174 | A48R | E1.1 | 0 | 502 | 670 | 1010 |
| VACWR175 | A49R | E1.2 | 0 | 296 | 310 | 401 |
| VACWR176 | A50R | E1.2 | 0 | 21 | 326 | 398 |
| VACWR177 | A51R | E1.1 | 0 | 246 | 954 | 908 |
| VACWR178 | A52R | E1.2 | 0 | 437 | 103 | 93 |
| VACWR179 | A53R | PR | 0 | 0 | 50 | 75 |
| VACWR180 | A55R | E1.2 | 0 | 29 | 113 | 116 |
| VACWR181 | A56R | E1.2 | 0 | 310 | 927 | 876 |
| VACWR181.5 | 269 | E1.2 | 0 | 0 | 336 | 338 |
| VACWR182 | A57R | E1.2 | 0 | 1484 | 1855 | 1780 |
| VACWR183 | B1R | E1.2 | 0 | 445 | 677 | 805 |
| VACWR184 | B2R | E1.2 | 0 | 1269 | 568 | 891 |
| VACWR185 | B3R | E1.1 | 0 | 1095 | 1771 | 2252 |
| VACWR186 | B4R | PR | 0 | 1 | 32 | 28 |
| VACWR187 | B5R | E1.2 | 0 | 63 | 619 | 665 |
| VACWR188 | B6R | E1.2 | 0 | 2557 | 4006 | 1995 |
| VACWR189 | B7R | PR | 0 | 84 | 18 | 21 |
| VACWR190 | B8R | E1.1 | 0 | 3891 | 11729 | 15878 |
| VACWR191 | B9R | PR | 0 | 4 | 2 | 0 |
| VACWR192 | B10R | PR | 0 | 121 | 36 | 48 |
| VACWR193 | B11R | E1.2 | 0 | 159 | 119 | 67 |
| VACWR194 | B12R | E1.1 | 0 | 1931 | 1005 | 1234 |
| VACWR195 | B13R | E1.1 | 0 | 278 | 1412 | 1812 |
| VACWR196 | B15R | E1.2 | 0 | 1293 | 379 | 107 |
| VACWR197 | B16R | PR | 0 | 71 | 31 | 1 |
| VACWR198 | B17L | E1.2 | 0 | 41 | 63 | 62 |
| VACWR199 | B18R | E1.2 | 0 | 323 | 194 | 91 |
| VACWR200 | B19R | E1.1 | 0 | 3338 | 12171 | 9597 |
| VACWR201 | pseudogene | E1.2 | 0 | 0 | 66 | 57 |
| VACWR202 | B20R | E1.2 | 0 | 0 | 53 | 156 |
| VACWR203 | B20R | E1.2 | 0 | 249 | 671 | 810 |
| VACWR204 | no ortholog | E1.2 | 0 | 201 | 184 | 448 |
| VACWR204.5 | 264 | PR | 0 | 150 | 52 | 0 |
| VACWR205 | C12L | E1.2 | 0 | 167 | 346 | 314 |
| VACWR206 | C13L, C14L | PR | 0 | 0 | 5 | 30 |

^a^ The counts were normalized by: (i) the total mapped reads in the samples; (ii) the length (kbp) of the ORF; (iii) the very low background read number at time 0 of the ORFs (subtracted); (iv) read counts of diploid ORFs in the terminal repetitions (divided by 2).

^b^ The early genes required for DNA replication are underlined.

^c^ Both E1.1 and E1.2 are early. PR is post replicative. The temporal expression class was according to :Yang Z, Bruno DP, Martens CA, Porcella SF, Moss B (2010) Simultaneous high-resolution analysis of vaccinia virus and host cell transcriptomes by deep RNA sequencing. Proc Natl Acad Sci USA 107: 11513-11518.

*ORF with read-through and grouped with PR.

**Short ORFs in which the reads appeared continuous from the upstream ORF.
